# COVID‐19 in cancer patients: The impact of vaccination on outcomes early in the pandemic

**DOI:** 10.1002/cam4.6781

**Published:** 2023-12-08

**Authors:** Fareed Khawaja, Georgios Angelidakis, Adina Feldman, Vinod Ravi, Eric Woodman, Micah Bhatti, Ella Ariza‐Heredia, Peter Elhajj, Amy Spallone, Ying Jiang, Roy F. Chemaly

**Affiliations:** ^1^ Department of Infectious Diseases, Infection Control and Employee Health The University of Texas MD Anderson Cancer Center Houston Texas USA; ^2^ Data‐Driven Determinants for COVID‐19 Oncology Discovery Effort (D3CODE) Team The University of Texas MD Anderson Cancer Center Houston Texas USA; ^3^ Department of Sarcoma Medical Oncology, Division of Cancer Medicine The University of Texas MD Anderson Cancer Center Houston Texas USA; ^4^ Department of Genomic Medicine, Division of Cancer Medicine The University of Texas MD Anderson Cancer Center Houston Texas USA; ^5^ Department of Laboratory Medicine The University of Texas MD Anderson Cancer Center Houston Texas USA

**Keywords:** cancer, COVID‐19, immunocompromised, SARS‐CoV‐2, vaccination

## Abstract

**Background:**

With the rapid evolution of the severe acute respiratory syndrome coronavirus 2 (SARS‐CoV‐2) pandemic, the development of effective and safe vaccines was of utmost importance to protect vulnerable individuals, including cancer patients. Studies comparing the clinical outcomes of cancer patients with or without vaccination against coronavirus disease 2019 (COVID‐19) have not demonstrated clear benefit. We aimed to determine the protective effects of COVID‐19 vaccination by comparing vaccinated and unvaccinated cancer patients after the initial phase of vaccine roll‐out and to identify risk factors associated with hospitalization, severe COVID‐19, and 30‐day COVID‐19 attributable mortality.

**Methods:**

We performed a retrospective cohort study of cancer patients with COVID‐19 diagnosed by polymerase chain reaction on nasal swabs between January 1, 2021 and July 30, 2021. Outcomes of interest included hospitalization, severe COVID‐19, and 30‐day COVID‐19 attributable mortality. Univariate and multivariate analyses were performed to identify factors associated with clinical outcomes, using vaccination status as a variable of interest in all models.

**Results:**

Key risk factors, such as age ≥ 60 years; comorbidities including diabetes mellitus, heart failure, and lung diseases; and specific cancer types (leukemia and lymphoma) were independently associated with hospital admission for COVID‐19, severe COVID‐19, and 30‐day COVID‐19 attributable mortality in cancer patients regardless of their vaccination status. Vaccinated patients were protected against severe COVID‐19 but with no impact on hospitalization or mortality due to COVID‐19.

**Conclusion:**

Our study highlights a significant benefit of COVID‐19 vaccination for cancer patients—specifically its protection against severe COVID‐19.

## 1 INTRODUCTION

With the rapid evolution of the severe acute respiratory syndrome coronavirus 2 (SARS‐COV‐2) pandemic, the development of effective and safe vaccines with immediate deployment became a key strategy to reduce the spread of the virus and protect against severe infections and death.^1^ The first two vaccines against coronavirus disease 2019 (COVID‐19), Pfizer‐BioNTech's BNT162b2 and Moderna's mRNA‐1273 vaccines, were approved on December 11 and 18, 2020, respectively.^2^ The emergency use authorizations were based on Phase 3 data that reported >94% and 100% efficacy in preventing symptomatic COVID‐19 and severe infections, respectively.^3^, ^4^ However, timing of COVID‐19 vaccinations and the number of needed doses in special populations, such as immunocompromised individuals and cancer patients, were uncertain due to lack of clinical data.^5^

Immunocompromised patients with cancer and prior transplant are vulnerable to breakthrough infections and complications related to COVID‐19.^6^, ^7^, ^8^, ^9^ Severe COVID‐19, use of mechanical ventilation, and overall mortality are reportedly greater among cancer patients than among the general population.^6^, ^7^, ^8^, ^9^, ^10^, ^11^, ^12^ Multiple risk factors have been identified, such as age, use of active chemotherapy, and type of cancer—with patients with hematologic malignancies (HM) having worse clinical outcomes than patients with solid tumors.^13^, ^14^ Despite the potential risk of breakthrough infections among cancer patients after COVID‐19 vaccination when compared to healthy controls, this preventive strategy may protect cancer patients against severe COVID‐19 and may improve overall survival.^15^, ^16^

Vaccinating cancer patients against influenza has proved safe and somewhat effective^15^ and provided a basis to promote vaccination of cancer patients after the approval of the BNT162b2/Pfizer–BioNTech and mRNA‐1273/Moderna mRNA COVID‐19 vaccines. However, many studies measuring humoral responses after COVID‐19 vaccination in cancer patients raised concerns about reduced efficacy.^17^, ^18^, ^19^ Reduced efficacy based on humoral response was more pronounced among HM patients compared with solid tumor patients^19^; this poor serologic response and underlying immunocompromised status likely contributed to higher rates of breakthrough infections when compared to healthy individuals.^20^, ^21^, ^22^, ^23^

Breakthrough COVID‐19 in vaccinated cancer patients has been previously associated with poor outcomes.^16^, ^19^, ^24^ Underlying HM, immunosuppressive drugs use, and prior hematopoietic cell transplant (HCT) were major risk factors for breakthrough infections.^16^, ^24^, ^25^, ^26^ Among the cohorts with varying immunocompromising conditions, breakthrough COVID‐19 was associated with greater rates of hospitalization and in‐hospital death.^12^, ^27^ Similar findings were noted when HM patients with breakthrough infections were compared with patients without cancer.^23^ Among solid organ transplant recipients, breakthrough COVID‐19 in fully vaccinated patients was not clearly associated with worse outcomes.^28^ On the other hand, earlier studies focusing on cancer patients did not demonstrate any clinical benefit, such as reduction in COVID‐19–related mortality among cancer patients who underwent COVID‐19 vaccination.^16^

We aimed in this large cohort study to identify risk factors associated with COVID‐19–related outcomes such as rates of hospitalization, severe COVID‐19, and 30‐day COVID‐19 attributable mortality and to determine if any protective effects of COVID‐19 vaccination in cancer patients compared with unvaccinated cancer patients after the initial phase of vaccines rollout for immunocompromised patients during the Alpha and Delta waves or surges.

## 2 METHODS

### 2.1 Study design

We performed a retrospective cohort study that included consecutive cancer patients who were diagnosed with COVID‐19 via polymerase chain reaction on nasal swabs from January 1, 2021 to July 30, 2021 (during the Alpha and Delta waves), regardless of their vaccination status at the time of diagnosis. During this time period, the CDC recommended 2 doses of either mRNA vaccine (Pfizer–BioNTech or Moderna) or 1 dose of the adenovirus vector‐based vaccine (Janssen) for immunocompromised patients; our study was conducted before the recommendation of booster doses. All cancer patients undergoing active follow‐up in our institution at any stage of treatment were included. Patients were excluded if they had no diagnosis of cancer or had precancerous syndrome (e.g., myelodysplastic syndrome), had no recorded date of vaccination despite documentation of vaccination before infection, or were originally diagnosed with COVID‐19 outside of the study period. Only the first episode of COVID‐19 was included for analysis, and multiple episodes per patient were excluded. Patients' demographics, vaccination history, admission data, laboratory data, oncologic history, and other comorbidities were collected. The study objectives were to determine COVID‐19–related complications, including severe COVID‐19, hospitalization for COVID‐19, and 30‐day COVID‐19 attributable mortality. Severe COVID‐19 was defined based on requirement of oxygen supplementation for hypoxia within 30 days of COVID‐19 diagnosis, in line with the National Institutes of Health's (NIH) definition of severe infection.^29^ Vaccination status was categorized as unvaccinated, partially vaccinated, or fully vaccinated at the time of COVID‐19. Partially vaccinated was defined as having received only 1 dose of either mRNA vaccine before infection or being diagnosed with COVID‐19 within 14 days of the second dose. Fully vaccinated was defined as having received 2 doses of either mRNA vaccine or 1 dose of the adenovirus vector‐based vaccine more than 14 days before COVID‐19. This study was performed after our institutional review board's approval, and a waiver of consent was granted.

### 2.2 Data collection

Patients with COVID‐19 were identified using our institution's infection control database. Demographics, comorbidities, laboratory data within 7 days of diagnosis, and COVID‐19 directed therapies within 14 days of diagnosis were collected using Palantir Foundry (Syntropy) as previously described.^30^ Oncologic data were collected via our tumor registry. The study objectives and patients' vaccination status were determined via review of patients' electronic medical records.

### 2.3 SARS‐CoV‐2 testing and protocols

SARS‐CoV‐2 testing was performed using one of two real‐time polymerase chain reaction assays available at our institution: the Cobas SARS‐CoV‐2 assay performed on the Cobas 6800 system (Roche Diagnostics) or the Abbott SARS‐CoV‐2 test performed on the m2000 system. Patients were tested if they were symptomatic (cough, fever, signs of pneumonia on imaging, rhinorrhea, or shortness of breath), or before admission or procedures if asymptomatic. Vaccination status was not considered when testing patients for COVID‐19. Patients were treated with available therapeutics for COVID‐19 based on its severity. These included remdesivir and steroids for patients with hypoxia in addition to tocilizumab or anakinra for patients with severe hypoxia.

### 2.4 Statistical analysis

For the purpose of analyzing the study objectives, outcomes were considered binary variables. Vaccination status was also analyzed as a binary variable; partially vaccinated patients were considered unvaccinated for the purpose of the analysis. To identify the independent predictors of the patient outcomes and evaluate the independent impact of vaccination on the outcomes, we carried out logistic regression model analysis with propensity score adjustment on each outcome. First, propensity score was generated using logistic regression analysis by modeling the probability of receiving vaccination for each patient. This logistic regression model included all the variables with a *p*‐value ≤0.25 on their univariate association analyses with vaccination (Figure S1). Then when we carried out a logistic regression to analyze the association with each patient outcome (COVID‐19‐related hospitalization, all‐cause 30‐day mortality, and severe VCOVID‐19), respectively. This propensity score was incorporated into the logistic regression analysis using stabilized inverse probability of treatment weighted (IPTW) method to reduce the baseline imbalance between patients who received vaccination and those who did not. Treatment for COVID‐19 was given based on severity using a standardized algorithm during the study period; these variables were not included in our analysis.

## 3 RESULTS

### 3.1 Patient cohort

We identified a total of 963 patients with COVID‐19 from the infection control database. From those, 155 patients were excluded for the following reasons: 51 were diagnosed outside the study period, 82 had no history of cancer or premalignant condition, 4 were considered duplicate as they tested positive for SARS‐CoV‐2 repeatedly within 14 days of the first positive test, 5 had no documented date of COVID‐19 diagnosis, and 13 had unknown COVID‐19 vaccination dates. In addition, 1 patient was admitted at an outside hospital for COVID‐19, and we could not ascertain the severity of COVID‐19. Overall, 808 cancer patients with COVID‐19 were included for analysis; 207 with HM and 601 with solid tumors. Most patients were unvaccinated at time of COVID‐19 diagnosis (*n* = 635; 79%). Among the 114 fully and 59 partially vaccinated patients with breakthrough COVID‐19, most had received BNT162b2/Pfizer–BioNTech COVID‐19 vaccines (Table 1).

**TABLE 1:** Characteristics of vaccinated and unvaccinated cancer patients with COVID‐19.

Variables	Total (*N* = 808)	Vaccinated (*N* = 114)	Unvaccinated (*N* = 694)	*p* Value
Age, median (range), years	62 (2–97)	66 (28–97)	61 (2–94)	<0.0001
Gender, no. (%)				0.1580
Male	397 (49)	63 (55)	333 (48)	
Female	416 (51)	51 (45)	361 (52)	
Ethnicity, no. (%)				0.3774
Hispanic or Latino	160 (20)	19 (17)	141 (20)	
Not Hispanic or Latino	619 (76)	93 (82)	526 (76)	
Declined to answer/unknown	29 (4)	2 (2)	27 (4)	
Race, no. (%)				0.7225
White or Caucasian	613 (75)	88 (77)	520 (75)	
American Indian or Alaskan native	7 (1)	0 (0)	7 (1)	
Asian	33 (4)	6 (5)	27 (4)	
Black or African‐American	68 (8)	8 (7)	60 (9)	
No answer	10 (1)	0 (0)	10 (13)	
Other	82 (10)	12 (11)	70 (10)	
Smoking status, no. (%)				
Former smoker	289 (36)	47 (41)	240 (35)	0.1715
Never smoker	490 (60)	65 (57)	422 (61)	0.4704
Current smoker	23 (3)	2 (2)	21 (3)	0.7594
Unknown smoking status	11 (1)	0 (0)	11 (2)	0.3791
Noncancer comorbidities, no. (%)				
Hypertension	453 (56)	78 (68)	375 (54)	0.0043
Diabetes mellitus	238 (30)	34 (30)	204 (29)	0.9122
Heart failure	49 (6)	12 (11)	37 (5)	0.0529
COPD	62 (8)	11 (10)	51 (7)	0.4458
Asthma	102 (13)	18 (16)	84 (12)	0.2866
Lung fibrosis	20 (2)	3 (3)	17 (2)	0.7538
HIV	5 (<1)	2 (2)	3 (<1)	0.1481
Chronic kidney disease stage 3–5	154 (19)	22 (19)	132 (19)	1.0000
End‐stage renal disease	14 (2)	2 (2)	12 (2)	1.0000
BMI				
BMI continuous variable, median (range)	28 (11–61)	29.2 (16.1–55.3)	28 0.5 (11.1–61.7)	0.9428
BMI category, no, (%)				0.2695
<18.5	28 (4)	2 (2)	26 (4)	
18.5 to −<25.0	185 (23)	21 (18)	161 (24)	
25.0 to <30	252 (32)	45 (39)	207 (31)	
30 to <40	273 (35)	40 (35)	233 (35)	
≥40	52 (7)	6 (5)	46 (7)	
Type of cancer, no. (%)				1.000
Solid tumor	601 (74)	85 (74)	516 (74)	
Hematologic malignancy	207 (26)	29 (26)	178 (26)	
Type of primary cancer, no. (%)				
Breast	137 (17)	19 (17)	118 (17)	1.0000
Central nervous system	10 (1)	1 (1)	9 (1)	1.0000
Eye, ears, nose, and throat	57 (7)	4 (4)	53 (8)	0.1639
Endocrine	20 (2)	2 (2)	18 (3)	1.0000
Gastrointestinal	120 (15)	20 (18)	99 (14)	0.3917
Genitourinary	84 (10)	14 (12)	70 (11)	0.5071
Gynecologic	35 (4)	7 (6)	28 (4)	0.3184
Lung/thoracic	37 (5)	8 (7)	29 (4)	0.2214
Melanoma	40 (5)	6 (5)	34 (5)	0.8168
Musculoskeletal/sarcoma	32 (4)	2 (2)	30 (4)	0.2972
Nonmelanoma skin	22 (3)	1 (1)	21 (3)	0.3460
Unknown primary	5 (<1)	0 (0)	5 (1)	1.0000
Multiple myeloma	24 (3)	1 (1)	23 (3)	0.2331
Lymphoma	103 (13)	22 (19)	81 (12)	0.0328
Leukemia	83 (10)	7 (6)	76 (11)	0.1348
Prior hematopoietic cell transplant, no. (%)	60 (7)	3 (3)	57 (8)	0.0334
Vaccine manufacturer, no. (%)				
Janssen/Ad26.COV2.S		8 (<1)	Not applicable	
Moderna/mRNA‐1273		25 (3)	Not applicable	
Pfizer‐BioNTech/BNT162b2		81 (10)	Not applicable	
Time from vaccination to infection in fully vaccinated patients, median (range), days		102 (15–183)	Not applicable	‐
COVID‐19–directed treatments, no. (%)				
Bamlanivimab	35 (4)	0 (0)	35 (5)	0.0101
Casirivimab‐imdevimab	25 (3)	7 (6)	18 (3)	0.0709
Remdesivir	154 (19)	25 (22)	129 (19)	0.4398
Steroids	210 (26)	32 (28)	178 (26)	0.5669
Convalescent plasma	11 (1)	0 (0)	11 (2)	0.3791
Tocilizumab	22 (3)	3 (3)	19 (3)	1.0000
Anakinra	2 (<1)	0 (0)	2 (<1)	1.0000
COVID‐19–related outcomes, no. (%)				
Admission for COVID‐19	242 (30)	32 (28)	210 (30)	0.5810
Severe COVID‐19^a^	132 (16)	14 (12)	118 (17)	0.1694
High‐flow nasal cannula/mechanical ventilation^a^	70 (9)	8 (7)	62 (9)	0.3714
In‐hospital mortality	32 (4)	3 (3)	29 (4)	0.2952
30‐day all‐cause mortality	39 (5)	6 (5)	33 (5)	0.8168
30‐day COVID‐19 attributable mortality	25 (3)	3 (3)	22 (3)	1.0000

Abbreviations: BMI, body mass index; COPD, chronic obstructive pulmonary disease; HIV, human immunodeficiency virus.

^a^ *N* = 807 due to lack of data on severity of COVID‐19.

### 3.2 COVID‐19–related outcomes in vaccinated and unvaccinated patients with cancer

**FIGURE 1:**
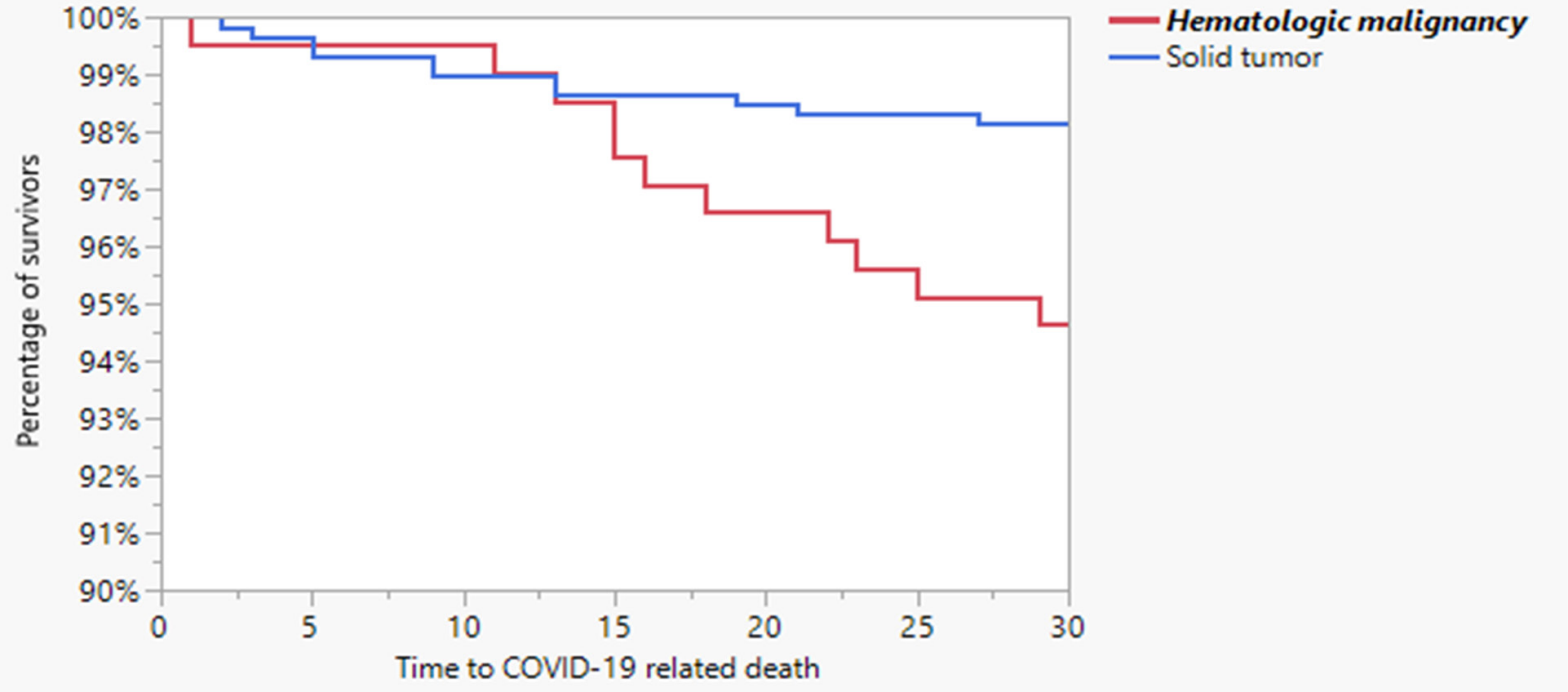
Survival curve (Kaplan–Meier) comparing 30‐day COVID‐19 attributable mortality in patients with hematologic malignancies and solid tumors (Log rank: *p* = 0.0089).

Tables 1 and 2 depict the baseline characteristics of the cohort, stratified by vaccination status and whether hospitalized or not. Overall, 30% were hospitalized, 16% had severe COVID‐19, 9% required high‐flow nasal cannula or mechanical ventilation, and 5% died within 30 days of COVID‐19 diagnosis. Compared with unvaccinated patients, vaccinated patients were older, more likely to have hypertension and lymphoma, and less likely to have undergone HCT (Table 1). In addition, vaccinated patients had a higher absolute monocyte count (AMC) and absolute neutrophil count (ANC) (Table S1). More unvaccinated patients than vaccinated patients received bamlanivimab for their COVID‐19; otherwise the use of antiviral and anti‐inflammatory therapies were similar between vaccinated and unvaccinated patients. COVID‐19–related outcomes were similar in vaccinated and unvaccinated patients (Table 1). 30‐day mortality attributable to COVID‐19 was significantly higher in HM patients when compared to patients with solid tumors (Figure 1).

**TABLE 2:** Characteristics of COVID‐19–related hospitalization in cancer patients.

Variable		Admitted for COVID‐19 (*N* = 242)	Not Admitted for COVID‐19 (*N* = 566)	*p* Value
Age, median (range) years	62 (2–97)	64 (6–94)	61 (2–97)	0.0003
Gender, no. (%)				
Male	397 (49)	124 (52)	270 (48)	0.2811
Female	416 (51)	114 (48)	296 (52)	
Ethnicity, no. (%)				0.1220
Hispanic or Latino	160 (20)	175 (72)	444 (78)	
Not Hispanic or Latino	619 (76)	55 (23)	105 (19)	
Declined to answer/unknown	29 (4)	12 (5)	17 (3)	
Race, no. (%)				
White or Caucasian	613 (75)	171 (71)	436 (77)	0.0542
American Indian or Alaskan native	7 (1)	1 (<1)	6 (1)	
Asian	33 (4)	11 (5)	21 (4)	
Black or African‐American	68 (8)	17 (7)	51 (9)	
No answer	10 (1)	5 (2)	5 (1)	
Other	82 (10)	35 (15)	47 (8)	
Smoking status, no. (%)				
Former smoker	289 (36)	98 (41)	188 (33)	0.0444
Never smoker	490 (60)	135 (56)	351 (62)	0.1354
Current smoker	23 (3)	6 (3)	17 (3)	0.8196
Unknown smoking status	11 (1)	1 (<1)	10 (2)	0.1888
Noncancer comorbidities, no. (%)				
Hypertension	453 (56)	165 (68)	288 (51)	<0.0001
Diabetes mellitus	238 (30)	103 (43)	135 (24)	<0.0001
Heart failure	49 (6)	32 (13)	17 (3)	<0.0001
COPD	62 (8)	40 (16)	22 (4)	<0.0001
Asthma	102 (13)	35 (14)	67 (12)	0.4175
Lung fibrosis	20 (2)	8 (3)	12 (2)	0.3256
HIV	5 (<1)	1 (4)	4 (1)	1.0000
Chronic kidney disease stage 3–5	154 (19)	87 (36)	67 (12)	<0.0001
End‐stage kidney disease	14 (2)	9 (4)	5 (1)	0.0072
BMI continuous variable, median (range), years		27 (13–61)	29 (11–60)	0.0004
BMI category	28 (11–61)			
<18.5		14 (5)	14 (3)	
18.5–<25.0	28 (4)	71 (30)	111 (20)	
25.0–<30	185 (23)	71 (30)	181 (33)	
30–<40	252 (32)	72 (30)	201 (37)	
≥40	273 (35)	12 (5)	40 (7)	
Type of cancer, no. (%)	52 (7)			<0.0001
Solid tumor		145 (60)	456 (81)	
Hematologic malignancy	601 (74)	97 (40)	110 (19)	
Type of primary cancer, no. (%)	207 (26)			
Breast		22 (9)	115 (20)	<0.0001
Central nervous system	137 (17)	1 (<1)	9 (2)	0.2962
Eye, ears, nose, and throat	10 (1)	13 (5)	44 (8)	0.1754
Endocrine	57 (7)	5 (2)	15 (3)	0.8060
Gastrointestinal	20 (2)	38 (16)	81 (14)	0.5875
Genitourinary	120 (15)	19 (8)	65 (11)	0.1648
Gynecologic	84 (10)	8 (3)	27 (5)	0.4512
Lung/thoracic	35 (4)	15 (6)	22 (4)	0.2727
Melanoma	37 (5)	10 (4)	30 (5)	0.5964
Musculoskeletal/sarcoma	40 (5)	8 (3)	24 (4)	0.6938
Nonmelanoma skin	32 (4)	4 (2)	18 (3)	0.3435
Unknown primary	22 (3)	1 (<1)	4 (1)	1.0000
Multiple myeloma	5 (<1)	9 (4)	15 (3)	0.3749
Lymphoma	24 (3)	43 (18)	60 (11)	0.0055
Leukemia	103 (13)	46 (19)	37 (7)	<0.0001
Prior hematopoietic cell transplant, no. (%)	83 (10)	32 (13)	28 (5)	0.0001
Vaccination status, no. (%)				
Partially vaccinated		16 (7)	43 (8)	0.6613
Fully vaccinated		32 (13)	82 (14)	0.6609
Unvaccinated		194 (80)	441 (78)	0.5129
Type of vaccine received, no. (%)				0.2220
Janssen/Ad26.COV2.S		2 (1)	43 (8)	
Moderna/mRNA‐1273		3 (1)	82 (14)	
Pfizer‐BioNTech/BNT162b2		27 (11)	441 (78)	
Time from vaccination to infection in fully vaccinated patients, median (range), days		80 (16–183)	107 (15–176)	0.1689
COVID‐19–directed therapy, no. (%)				
Bamlanivimab	35 (4)	12 (5)	23 (4)	0.5719
Casirivimab‐imdevimab	25 (3)	7 (3)	18 (3)	1.0000
Remdesivir	154 (19)	152 (63)	2 (<1)	<0.0001
Steroids	210 (26)	135 (56)	75 (13)	<0.0001
Convalescent plasma	11 (1)	10 (4)	1 (<1)	<0.0001
Tocilizumab	22 (3)	22 (18)	0 (0)	<0.0001
Anakinra	2 (<1)	2 (2)	0 (0)	0.0880
COVID‐19–related outcomes, no. (%)				
Severe COVID‐19^a^	132 (16)	127 (55)	0 (0)	<0.0001
High‐flow nasal cannula/mechanical ventilation^a^	70 (9)	70 (30)	0 (0)	<0.0001
30‐day all‐cause mortality	39 (5)	24 (10)	1 (<1)	<0.0001
30‐day COVID‐19 attributable mortality	25 (3)	24 (10)	1 (<1)	<0.0001

Abbreviations: BMI, body mass index; COPD, chronic obstructive pulmonary disease; HIV, human immunodeficiency virus.

^a^ *N* = 807 due to lack of data on severity of COVID‐19.

#### 3.2.1 Hospitalization

On univariate analysis, risk factors for hospitalization included older age, former smoking status, and multiple comorbidities such as history of hypertension, diabetes mellitus, heart failure, chronic obstructive pulmonary disease (COPD), Stage 3–5 chronic kidney disease (CKD), end‐stage kidney disease, and having lower body mass index (BMI) (Table 2). In addition, patients with HM were more likely to be hospitalized; in particular, patients who had undergone HCT and/or had leukemia or lymphoma. Univariate analysis of laboratory correlatives is depicted in Table S2.

The multivariate analysis using logistic regression model with propensity score adjustment (excluding laboratory variables) included 787 patients whose BMI measurements were available at the time of COVID‐19 diagnosis. Independent predictors of hospitalization included history of heart failure, diabetes mellitus, COPD, Stage 3–5 CKD, lymphoma, and leukemia (Table 3). A high BMI was protective against hospitalization.

**TABLE 3:** Multivariate logistic regression model of hospitalization and independent impact of vaccination.

Variable	Odds ratio	95% CI	*p* value
All laboratory variables excluded (*N* = 787)			
Diabetes mellitus	2.01	1.40 to 2.90	<0.001
Heart failure	2.60	1.34 to 5.06	0.005
COPD	3.66	2.02 to 6.66	<0.0001
CKD stage 3–5	3.01	1.99 to 4.54	<0.0001
BMI ≥30	0.62	0.43 to 0.89	0.009
Lymphoma	2.74	1.71 to 4.38	<0.0001
Leukemia	3.30	1.95 to 5.58	<0.0001
Vaccination	0.66	0.39 to 1.11	0.12
Laboratory variables included* (*N* = 741)			
Diabetes mellitus	1.66	1.12 to 2.45	0.011
Heart failure	2.49	1.20 to 5.20	0.015
COPD	4.65	2.43 to 8.92	<0.0001
CKD stage 3–5	2.29	1.46 to 3.60	<0.001
Leukemia	2.62	1.57 to 4.37	<0.001
Lymphoma	2.89	1.66 to 5.03	<0.001
Albumin <3.5 g/dL	6.99	4.20 to 11.64	<0.0001
AMC <0.45 k/μL	1.81	1.24 to 2.65	0.002
AST >28 U/L	2.22	1.52 to 3.23	<0.0001
Vaccination	0.59	0.33 to 1.04	0.068

Abbreviations: AST, aspartate transaminase; CKD, chronic kidney disease; COPD, chronic obstructive pulmonary disease; HIV, human immunodeficiency virus.

* Included laboratory variables: albumin, creatinine, absolute neutrophil count, absolute lymphocyte count, absolute monocyte count, and aspartate transaminase.

The multivariate analysis that included laboratory values encompasses 741 patients who had albumin, ANC, AMC, absolute lymphocyte count (ALC), and aspartate transaminase (AST) measured at time of COVID‐19 diagnosis. Independent predictors of hospitalization included heart failure, diabetes mellitus, COPD, and Stage 3–5 CKD (Table 3). Other factors such as having leukemia, lymphoma, a reduced albumin, a reduced AMC, and an elevated AST were also independently associated with COVID‐19–related hospitalization. Vaccination was not protective against COVID‐19–related hospitalization in either model, although a trend was seen in the latter (*p* = 0.068).

#### 3.2.2 Severe COVID‐19

The analysis with severe COVID‐19 as the outcome measure included 807 patients. On univariate analysis, older age, smoking history, and comorbidities such as hypertension, diabetes mellitus, heart failure, COPD, lung fibrosis, Stage 3–5 CKD, and end‐stage kidney disease were associated with severe COVID‐19 (Table 4). In addition, having an underlying HM, such as leukemia or lymphoma, was associated with severe COVID‐19 compared with patients with solid tumors. Having no smoking history was protective against severe COVID‐19. Abnormal laboratory findings associated with severe COVID‐19 included reduced white blood cell count, reduced albumin, reduced ALC, and reduced AMC (Table S2).

**TABLE 4:** Characteristics of cancer patients with or without severe COVID‐9.

Variable	Severe COVID‐19 (*N* = 132)	Non‐severe COVID‐19 (*N* = 675)	*p* Value
Age, median (range), years	68 (10–92)	61 (2–97)	<0.0001
Gender, no. (%)			0.5681
Male	68 (52)	327 (48)	
Female	64 (48)	348 (52)	
Ethnicity, no. (%)			0.0923
Hispanic or Latino	96 (73)	522 (77)	
Not Hispanic or Latino	27 (20)	133 (20)	
Declined to answer/unknown	9 (7)	20 (3)	
Race, no. (%)			0.0620
White or Caucasian	91 (69)	516 (76)	
American Indian or Alaskan native	0 (0)	7 (1)	
Asian	8 (6)	25 (4)	
Black or African‐American	10 (8)	58 (9)	
No answer	0 (0)	1 (<1)	
Other	19 (14)	63 (9)	
Smoking status, no. (%)			
Former smoker	64 (48)	222 (33)	0.0010
Never smoker	65 (49)	422 (63)	0.0048
Current smoker	2 (2)	21 (3)	0.4041
Unknown smoking status	1 (1)	10 (1)	1.000
Noncancer comorbidities, no. (%)			
Hypertension	101 (77)	351 (52)	<0.0001
Diabetes mellitus	66 (50)	172 (25)	<0.0001
Heart failure	24 (18)	25 (4)	<0.0001
COPD	32 (24)	29 (4)	<0.0001
Asthma	22 (17)	80 (12)	0.1509
Lung fibrosis	8 (6)	12 (2)	0.0092
HIV	0 (0)	5 (1)	1.0000
Chronic kidney disease stage 3–5	45 (34)	109 (16)	<0.0001
End‐stage renal disease	6 (5)	8 (1)	0.0166
BMI continuous variable, median (range)	27.95 (17.3–61.74)	28.79 (11.13–60.7)	0.4313
BMI category, no. (%)			0.9120
<18.5	4 (3)	24 (4)	
18.5–<25.0	34 (26)	148 (23)	
25.0–<30	42 (32)	209 (32)	
30–<40	42 (32)	231 (35)	
≥40	9 (7)	43 (7)	
Type of cancer, no. (%)			
Solid tumor	80 (61)	520 (77)	Reference
Hematologic malignancy	52 (39)	155 (23)	0.0002
Type of primary cancer, no. (%)			
Breast	15 (11)	122 (18)	0.751
Central nervous system	0 (0)	10 (1)	0.3810
Eye, ears, nose, and throat	9 (7)	47 (7)	1.0000
Endocrine	3 (2)	17 (3)	1.0000
Gastrointestinal	14 (10)	105 (16)	0.1788
Genitourinary	12 (9)	72 (11)	0.7550
Gynecologic	5 (4)	30 (4)	1.0000
Lung/thoracic	11 (8)	26 (14)	0.0373
Melanoma	4 (3)	36 (5)	0.3793
Musculoskeletal/sarcoma	1 (8)	31 (5)	0.0469
Nonmelanoma skin	4 (3)	18 (3)	0.7713
Unknown primary	1 (1)	4 (1)	0.5916
Multiple myeloma	4 (3)	20 (3)	1.000
Lymphoma	27 (20)	76 (11)	0.0063
Leukemia	22 (17)	61 (9)	0.0118
Prior hematopoietic cell transplant, no. (%)	14 (11)	46 (7)	0.1454
Vaccination status, no. (%)			
Partially vaccinated	10 (8)	49 (7)	0.8559
Fully vaccinated	14 (10)	100 (15)	0.2217
Unvaccinated	108 (82)	526 (78)	0.3548
Vaccine manufacturer, no. (%)			
Janssen/Ad26.COV2.S	1 (1)	12 (2)	
Moderna/mRNA‐1273	6 (5)	45 (7)	
Pfizer‐BioNTech/BNT162b2	23 (17)	144 (21)	
Time from vaccination to infection in fully vaccinated patients, median (range), days	76 (16–145)	104 (15–183)	0.1352
COVID‐19–directed treatments, no. (%)			
Bamlanivimab	6 (5)	29 (4)	0.8179
Casirivimab‐imdevimab	2 (2)	23 (3)	0.4074
Remdesivir	106 (80)	49 (7)	<0.0001
Steroids	101 (77)	109 (16)	<0.0001
Convalescent plasma	8 (6)	3 (4)	<0.0001
Tocilizumab	22 (17)	0 (0)	<0.0001
Anakinra	2 (2)	0	0.0266

Abbreviations: BMI, body mass index; COPD, chronic obstructive pulmonary disease; HIV, human immunodeficiency virus.

The multivariate analysis using logistic regression model analysis with propensity score adjustment (excluding laboratory variables) included 807 patients; age ≥ 60 years, diabetes mellitus, heart failure, COPD, lymphoma, and leukemia were independent predictors of severe COVID‐19 (Table 5). Notably, patients who were fully vaccinated were protected against severe COVID‐19 in this model.

**TABLE 5:** Multivariate logistic regression model of severe COVID‐19 and independent impact of vaccination.

Variable	Odds Ratio	95% CI	*p* Value
All Laboratory variables excluded (*N* = 807)			
Age ≥ 60 years	2.13	1.34 to 3.40	0.002
Diabetes mellitus	2.30	1.51 to 3.51	0.0001
Heart failure	4.02	2.06 to 7.83	<0.0001
COPD	5.46	2.95 to 10.13	<0.0001
Dialysis dependent	3.88	1.03 to 14.71	0.046
Lymphoma	3.26	1.90 to 5.60	<0.0001
Leukemia	2.93	1.62 to 5.31	0.0004
Vaccination	0.45	0.22 to 0.91	0.026
Laboratory variables included* (*N* = 740)			
Age ≥ 60 years	2.07	1.22 to 3.49	0.007
Diabetes mellitus	1.90	1.18 to 3.07	0.008
Heart failure	3.86	1.79 to 8.35	<0.001
COPD	9.26	4.46 to 19.22	<0.0001
Lymphoma	3.71	2.00 to 6.90	<0.0001
Leukemia	2.59	1.36 to 4.94	0.004
Albumin <3.5 g/dL	7.80	4.55 to 13.35	<0.0001
AMC <0.45 K/μL	2.22	1.38 to 3.58	0 0.001
AST >28 U/L	3.49	2.15 to 5.68	<0.0001
Vaccination	0.39	0.18 to 0.85	0.018

Abbreviations: AMC, absolute monocyte count; AST, aspartate transaminase, COPD, chronic obstructive pulmonary disease.

* Included laboratory variables: albumin, absolute lymphocyte count, absolute monocyte count, alanine transaminase, and aspartate transaminase.

On multivariate analysis with laboratory variables included (740 patients included), age ≥ 60 years, diabetes mellitus, heart failure, COPD, lymphoma, and leukemia remained independently associated with severe COVID‐19 (Table 5). Reduced albumin, reduced AMC, and elevated AST were also associated with severe COVID‐19 (Table S3). Vaccination was protective against severe COVID‐19 in this model as well.

#### 3.2.3 30‐day COVID‐19 attributable mortality

The analysis with 30‐day COVID‐19 attributable mortality as the outcome measure included 794 patients. On univariate analysis, advanced age, having comorbidities such as heart failure, COPD, and Stage 3–5 CKD were associated with 30‐day COVID‐19 attributable mortality (Table 6). In addition, having an underlying HM, such as lymphoma or leukemia, was associated with higher mortality compared to patients with solid tumors (Figure 1 and Table 6). Multiple laboratory variables were associated with higher 30‐day COVID‐19 attributable mortality, including reduced WBC, albumin, ALC, AMC, higher lactate dehydrogenase, and higher C‐reactive protein (Table S4).

**TABLE 6:** Characteristics of cancer patients with or without 30‐day COVID‐19 attributable mortality.

Variables	Died due to COVID‐19 *N* = 25	Survived COVID‐19 *N* = 769	*p* value
Age/median years (range)	65 (49–91)	62 (3–97)	0.0301
Gender (%)			
Male	11 (44)	378 (49)	0.6868
Female	14 (56)	391 (51)	
Ethnicity (%)			0.2406
Hispanic or Latino	7 (28)	151 (20)	
Not Hispanic or Latino	16 (64)	592 (77)	
Declined to answer/unknown	2 (8)	26 (3)	
Race (%)			
White or Caucasian	16 (64)	582 (76)	0.7984
American Indian or Alaskan native	0 (0)	6 (1)	
Asian	2 (8)	31 (4)	
Black or Africa American	3 (12)	64 (8)	
No answer	0 (0)	9 (1)	
Other	4 (16)	77 (10)	
Smoking status (%)			
Former smoker	10 (40)	270 (35)	0.6720
Never smoker	15 (60)	467 (61)	1.0000
Current smoker	0 (0)	22 (3)	1.0000
Unknown smoking status	0 (0)	10 (1)	1.0000
Non cancer comorbidities (%)			
Hypertension	16 (64)	431 (56)	0.5402
Diabetes mellitus	8 (32)	223 (29)	0.8232
Heart failure	4 (16)	44 (6)	0.0581
Chronic obstructive pulmonary disease	5 (20)	56 (7)	0.0364
Asthma	3 (12)	97 (13)	1.0000
Lung fibrosis	0 (0)	20 (3)	1.0000
Human immunodeficiency virus	0 (0)	5 (1)	1.0000
Chronic kidney disease 3–5	11 (44)	141 (18)	0.0034
End stage renal disease	1 (4)	13 (2)	0.3634
Body mass index analysis (BMI)			
BMI continuous variable/median (range)	27 (18–62)	29 (11–60)	0.2047
BMI category			
<18.5	1 (4)	25 (3)	0.7667
18.5–<25.0	8 (32)	171 (23)	
25.0–<30	8 (32)	239 (32)	
30–<40	6 (24)	263 (35)	
40 and above	2 (8)	50 (7)	
Type of cancer (%)			
Solid tumor	11 (44)	578 (75)	Reference 0.0016
Hematologic malignancy	14 (56)	191 (25)	
Type of primary cancer (%)			
Breast	3 (12)	132 (17)	0.7857
Central nervous system	0 (0)	10 (1)	1.0000
Eye, ears, nose, and throat	3 (12)	54 (7)	0.4152
Endocrine	0 (0)	19 (2)	1.0000
Gastrointestinal	1 (4)	114 (15)	0.1574
Genitourinary	1 (4)	82 (11)	0.5032
Gynecologic	0 (0)	33 (4)	0.6191
Lung/Thoracic	1 (4)	36 (5)	1.0000
Melanoma	0 (0)	39 (5)	0.6288
Musculoskeletal/Sarcoma	1 (4)	30 (4)	1.0000
Non melanoma skin	0 (0)	22 (3)	1.0000
Unknown primary	0 (0)	5 (1)	1.0000
Multiple myeloma	0 (0)	24 (3)	1.0000
Lymphoma	7 (28)	96 (12)	0.0300
Leukemia	8 (32)	75 (10)	0.0025
Prior hematopoietic cell transplantation (%)	3 (12)	57 (7)	0.4270
Vaccination status (%)			
Partially vaccinated	1 (4)	58 (7)	1.000
Fully vaccinated	3 (12)	108 (14)	
Unvaccinated	21 (84)	603 (78)	
Of those vaccinated, what manufacturer (%)			
Janssen/Ad26.COV2.S	1 (4)	6 (8)	
Moderna/mRNA‐1273	0 (0)	24 (3)	
Pfizer‐BioNTech/BNT162b2	3 (12)	78 (10)	
Time from vaccination to infection in fully vaccinated patients/median days (range)	101 (62–145)	104 (15–183)	0.8771
COVID‐19 directed treatments (%)			
Bamlanivimab	0 (0)	35 (5)	0.6211
Casirivimab‐imdevimab	1 (4)	24 (3)	0.5562
Remdesivir	17 (68)	134 (17)	<0.0001
Steroids	19 (76)	187 (24)	<0.0001
Convalescent plasma	2 (8)	9 (1)	0.0440
Tocilizumab	7 (28)	15 (2)	<0.0001
Anakinra	2 (8)	0 (0)	0.0010

Abbreviations: BMI, body mass index; COPD, chronic obstructive pulmonary disease; HIV, human immunodeficiency virus.

The multivariate analysis using logistic regression model analysis with propensity score adjustment (excluding laboratory variables) included 808 patients; CKD stage 3–5, having lymphoma, and leukemia were independent predictors of 30‐day COVID‐19 attributable mortality (Table 7). On multivariate analysis with laboratory variables included (749 patients), having leukemia, elevated ALC and reduced albumin were independently associated with 30‐day mortality attributable to COVID‐19 (Table 7). Vaccination was not protective against 30‐day COVID‐19 attributable mortality in all models. On Kaplan–Meier survival analysis patients with HM had significantly higher 30‐day COVID‐19 attributable mortality when compared to patients with solid tumors (Figure 1).

**TABLE 7:** Multivariate logistic regression model of 30‐day COVID‐19 attributable mortality and the independent impact of vaccination.

Variable	Odds ratio	95% CI	*p* Value
All Laboratory variables excluded (*N* = 808)			
Chronic kidney disease 3–5	2.26	1.12 to 4.56	0.023
Lymphoma	2.49	1.11 to 5.62	0.028
Leukemia	2.39	1.01 to 5.70	0.049
Vaccination	1.28	0.53 to 3.09	0.59
Laboratory variables included* (*N* = 749)			
Leukemia	2.39	1.02 to 5.59	0.044
ALC > 1.2 k/μL	0.24	0.10 to 0.58	0.002
Albumin <3.5 gm/dL	4.59	2.33 to 9.03	<0.0001
Vaccination	1.12	0.45 to 2.79	0.81

Abbreviation: ALC, absolute lymphocyte count.

* Included laboratory variables: albumin, creatinine, absolute lymphocyte count, absolute monocyte count, and aspartate transaminase.

## 4 DISCUSSION

Our study shows that cancer patients who were fully vaccinated against COVID‐19 were protected from severe infections during the early Alpha and Delta COVID‐19 waves. Yet it is unclear if vaccination was able to prevent hospitalization or 30‐day attributable mortality to COVID‐19. We identified key risk factors, such as age ≥ 60 years and multiple comorbidities such as diabetes mellitus, heart failure, COPD, and CKD, that are independently associated with COVID‐19–related hospitalization, severe COVID‐19, and 30‐day COVID‐19 attributable mortality regardless of vaccination status. In addition, patients with HMs, such as lymphoma and leukemia, had worse outcomes, such as severe COVID‐19 and higher 30‐day COVID‐19 attributable mortality, compared with patients with solid tumors regardless of their vaccination status. We also report a high hospitalization rate but less severe COVID‐19 and low 30‐day mortality attributable to COVID‐19 among the whole cohort, regardless of the vaccination status.

Our study is in line with prior studies in immunocompetent patients and solid organ transplant recipients.^28^, ^31^, ^32^ A previous report by Schmidt et al. reported no benefit of vaccination among cancer patients.^16^ However, the study's endpoints did not include severe COVID‐19 but instead used surrogate markers such as intensive care unit admission and use of mechanical ventilation. In our study, we defined severe COVID‐19 as patients' requiring oxygen supplementation for hypoxia within 30 days of COVID‐19 diagnosis, in line with the NIH's definition.^29^ In addition, Schmidt et al. identified only 3% of patients who were fully vaccinated (14% of our cohort of cancer patients were fully vaccinated at the time of COVID‐19 diagnosis) and were unable to power their analysis to detect statistically significant differences between vaccinated and unvaccinated cancer patients.^16^

The initial Phase 3 trials to assess the efficacy of the BNT162b2/Pfizer‐BioNTech and mRNA‐1273/Moderna COVID‐19 mRNA vaccines reported a 95% and 94% efficacy for prevention of symptomatic infections, respectively.^3^, ^4^ Severe COVID‐19 was observed in only 1 patient who was fully vaccinated with BNT162b2/Pfizer‐BioNTech.^3^ However, real‐world experience during the first year after the release of the mRNA vaccines noted a higher rate of breakthrough COVID‐19 than observed in the Phase 3 trials.^22^, ^33^, ^34^, ^35^, ^36^ Multiple factors may explain this discrepancy, such as the rise of the new delta variant and other variants of concern^22^, ^35^, ^37^ during that time; increased infection rate among vaccinated elderly, immunocompromised, and cancer patients^33^, ^35^, ^36^; and waning immunity from the COVID‐19 vaccines over time.^20^, ^34^ Nevertheless, COVID‐19 vaccination prevented severe infections,^20^, ^22^, ^34^, ^35^ as we found in our study in fully vaccinated cancer patients.

Cancer patients are highly encouraged to undergo COVID‐19 vaccination despite the scarcity of data.^10^, ^38^ Wu et al. had previously demonstrated that COVID‐19 vaccination was associated with lower rates of COVID‐19 among cancer patients and reported a 58% overall vaccine clinical efficacy after 2 doses of the vaccine.^10^ However, patients with HM had the poorest vaccine clinical efficacy of 19%. The authors did not report on the protective impact of vaccination during infection. In our own study, we were able to demonstrate a reduction in severe COVID‐19 in vaccinated cancer patients, but 30‐day attributable mortality to COVID‐19 was similar between vaccinated and unvaccinated patients. Multiple confounders unique to our immunocompromised cancer patients may explain this discrepancy, such as advanced underlying cancer and transition to hospice care when further cancer treatment was thought to be futile.

Cancer patients have varied levels of immunosuppression. We found, as others have, that patients with HM are at greater risk for breakthrough COVID‐19 after vaccination and may have more COVID‐19–related complications than patients with solid tumors.^16^, ^24^, ^25^, ^39^ This is likely due to greater immunosuppression from underlying disease, chemotherapy or cellular therapy, or both—resulting in poor immunologic responses to COVID‐19 vaccination.^19^, ^26^, ^36^, ^40^, ^41^ Fendler et al. reported a low seroconversion rate (59%) in HM patients after 2 doses of either BNT162b2/Pfizer‐BioNTech or AZD1222/AstraZeneca COVID‐19 vaccine.^41^ This low response rate was particularly evident in HM patients receiving anti‐CD20 therapy.^19^ In our study, patients with underlying leukemia and lymphoma remained at higher risk for hospitalization, severe COVID‐19, and 30‐day COVID‐19 attributable mortality compared to patients with other type of cancer regardless of their vaccination status. To better elicit immunologic responses in HM patients, multiple booster doses have been recommended based on recent studies.^40^ Other strategies to prevent severe infections should be underscored in this vulnerable population, such as pre‐exposure prophylaxis with monoclonal antibodies when effective for the specific circulating SARS‐CoV‐2 variants or subvariants and early antiviral therapy in mildly symptomatic HM patients.

Our study has several limitations. We did not evaluate antibody responses to vaccination and SARS‐CoV‐2 sequencing in our cohort. Prior studies have measured humoral and cellular responses after COVID‐19 vaccination in immunocompromised patients,^19^, ^26^, ^36^, ^40^, ^41^ but no clear quantitative thresholds that may protect against SARS‐CoV‐2 infection have been validated. In addition, there is no clear consensus on the timing of antibody measurement after vaccination. During the study period, a team led by Musser et al at a neighboring center in Houston, Texas assessed the circulating COVID‐19 variants from a large pool of samples from the greater Houston area.^42^, ^43^ The predominant variant in the community was Alpha between January 2021 and May 2021,^43^ but cases of the Delta variant increased from April 2021 onward until almost 99.9% of tested samples were the Delta variant by July 2021.^42^ Our cancer patients may have had either variant given the study period. This suggests that COVID‐19–related complications may be linked to poor host response and less to the type of circulating variant early into the pandemic. Our study did not measure associations of COVID‐19 with chemotherapy or stage of cancer. Nevertheless, given the large number of patients included in this analysis, we believe that the results provide a general overview of the different cancer subtypes. Lastly, our study did not assess vaccine efficacy in preventing symptomatic or asymptomatic COVID‐19 in cancer patients, as we did not include a comparative group of cancer patients with no COVID‐19 who were vaccinated.

Our study highlights the benefit of COVID‐19 vaccination in our cancer patients—specifically its protection against severe COVID‐19. Despite vaccination, breakthrough COVID‐19 was associated with high morbidity and mortality, especially among severely immunocompromised patients with underlying HM. Optimization of vaccination in cancer patients and the use of additional doses of vaccines, and pre‐exposure prophylactic strategies such as COVID‐19 monoclonal antibodies for severely immunocompromised patients should be determined in future studies.

## AUTHOR CONTRIBUTIONS

**Fareed Khawaja:** Conceptualization (equal); data curation (lead); formal analysis (supporting); investigation (lead); project administration (lead); visualization (equal); writing – original draft (lead). **Georgios Angelidakis:** Data curation (supporting); investigation (supporting); methodology (supporting); software (supporting); writing – original draft (supporting); writing – review and editing (supporting). **Adina Feldman:** Data curation (equal); investigation (equal); software (equal); writing – review and editing (supporting). **Vinod Ravi:** Conceptualization (supporting); data curation (supporting); software (supporting); visualization (supporting); writing – review and editing (supporting). **Eric Woodman:** Data curation (supporting); software (lead); supervision (lead); writing – review and editing (supporting). **Micah Bhatti:** Investigation (supporting); project administration (supporting); writing – review and editing (supporting). **Ella Ariza‐Heredia:** Conceptualization (supporting); methodology (supporting); writing – review and editing (supporting). **Peter Elhajj:** Visualization (supporting); writing – original draft (supporting); writing – review and editing (supporting). **Amy Spallone:** Supervision (supporting); writing – review and editing (supporting). **Ying Jiang:** Data curation (supporting); methodology (lead); software (supporting); writing – review and editing (supporting). **Roy F. Chemaly:** Conceptualization (equal); formal analysis (supporting); investigation (supporting); methodology (supporting); project administration (equal); supervision (equal); writing – review and editing (lead).

## FUNDING INFORMATION

None.

## CONFLICT OF INTEREST STATEMENT

FK: Honorarium from Medscape for educational material. Research support from Eurofins‐Viracor; GA: None; AF: None; PE: None; MB: None; EAH: Research support from MERCK; AS: None; JY: None; RFC: serves as a consultant, speaker, or advisor for ADMA Biologics, Pulmotec, Janssen, Merck/MSD, Partner Therapeutics, Takeda, Shinogi, Genentech, Astellas, Tether, Adagio Therapeutics, Oxford Immunotec, Karius, Eurofins‐Viracor, Aicuris, Moderna, and Ansun Pharmaceuticals. He received research grants paid to his institution from Merck, Karius, AiCuris, Ansun Pharmaceuticals, Takeda, Genentech, Oxford Immunotec, and Eurofins‐Viracor.

## ETHICAL APPROVAL

This study was approved by the University of Texas MD Anderson Cancer Center IRB (Protocol PA15‐0002).

## Supporting information


Data S1.

## Data Availability

The data that support the findings of this study cannot be shared based on institutional restrictions.
