# Supplementary material for: COVID‐19 in cancer patients: The impact of vaccination on outcomes early in the pandemic
Source: Cancer Med. 2023 Dec 8;12(24):22006–22. doi: 10.1002/cam4.6781 (PMC10757141; doi:10.1002/cam4.6781)
Supplement: Supplementary file 1 — Data S1. [file CAM4-12-22006-s001.docx]

**Supplemental tables and figures**

**Figure 1: Standardized mean difference between vaccinated and unvaccinated for all observations and weighted observations**
**
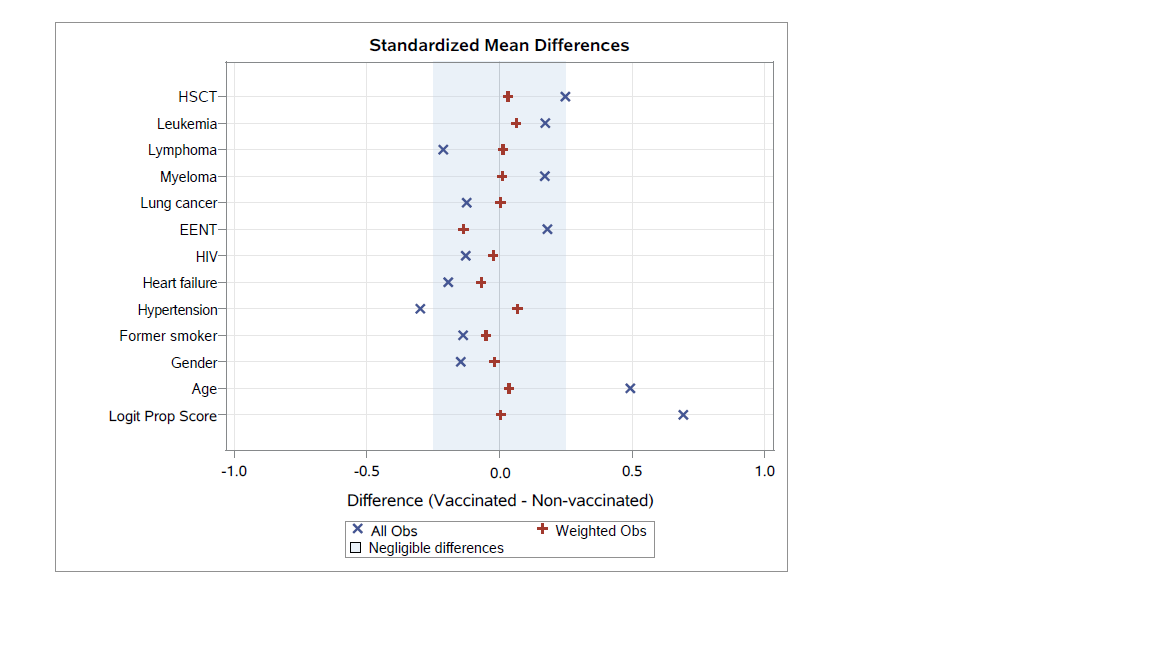
Note:** All standardized differences for the weighted observations within the recommended limits of –0.25 and 0.25, which are indicated by the shaded area.

**Abbreviations:** Logit Prop Score = Logit of the propensity score; Obs: observation/variable; EENT= Cancer of eye, ear, nose and throat; HIV= Human immunodeficiency virus; HSCT= Prior hematopoietic cell transplantation.

**Supplemental table 1. Laboratory values of vaccinated and unvaccinated cancer patients with COVID-19.**

|  | **Laboratory Values, Median (Range)** | | | | | | |
| --- | --- | --- | --- | --- | --- | --- | --- |
| **Laboratory Test** | **Total Cohort** | **Number Analyzed** | **Vaccinated** | **Number Analyzed** | **Unvaccinated** | **Number Analyzed** | ***P* Value** |
| White blood cell count, K/µL | 5.9  (0.1-737.5) | 774 | 6.1  (0.6-737.5) | 114 | 5.8  (0.1-310.8) | 660 | 0.0670 |
| Albumin, g/dL | 4.1  (1.7-5.1) | 753 | 4.1  (2.5-4.8) | 113 | 4.1  (1.7-5.1) | 640 | 0.2910 |
| Creatinine, mg/dL | 0.89  (0.24-12.73) | 774 | 0.93  (0.31-3.48) | 113 | 0.89  (0.24-12.73) | 661 | 0.0629 |
| Absolute neutrophil count, K/µL | 3.58  (0.02-59.98) | 771 | 3.93  (0.16-24.81) | 114 | 3.5  (0.02-59.98) | 657 | 0.0112* |
| Absolute lymphocyte count, K/µL | 1.15  (0.01-685.88) | 771 | 1.06  (0.05-685.88) | 114 | 1.17  (0.01-304.58) | 657 | 0.1712 |
| Absolute monocyte count, K/µL | 0.53  (0.01-6.72) | 771 | 0.58  (0.04-6.72) | 114 | 0.51  (0.01-6.29) | 657 | 0.0396* |
| Absolute CD4+ cell count, cells/µL | 343  (7-3396) | 74 | 548  (121-888) | 8 | 336  (7-3396) | 56 | 0.2911 |
| Absolute CD8+ cell count, Cells/µL | 365  (6-5754) | 74 | 395  (140-627) | 8 | 359  (6-5754) | 56 | 0.9192 |
| Procalcitonin, ng/dL | 0.13  (0.03-59.93) | 326 | 0.11  (0.03-36.72) | 41 | 0.14  (0.04-59.93) | 285 | 0.4127 |
| Fibrinogen, mg/dL | 474  (39-1074) | 371 | 218  (105-4239) | 92 | 467  (39-1074) | 279 | 0.1955 |
| Lactate dehydrogenase, U/L | 219  (92-4239) | 607 | 218  (105-4239) | 92 | 219  (92-3870) | 515 | 0.1698 |
| Alanine transaminase, U/L | 21  (0-1118) | 750 | 23  (5-142) | 113 | 21  (0-1118) | 637 | 0.5971 |
| Aspartate transaminase, U/L | 24  (9-2438) | 750 | 24  (10-116) | 113 | 24  (9-2438) | 637 | 0.9228 |
| Total bilirubin, mg/dL | 0.4  (0.2-7.3) | 629 | 0.4  (0.2-4.1) | 100 | 0.5  (0.2-7.3) | 529 | 0.3525 |
| Interleukin 6, pg/mL | 9.45  (2.2-92308.8) | 12 | 8.5 (-) | 1 | 9.5  (2.2-92308.8) | 11 | 0.6639 |
| C-reactive protein, mg/L | 32.98  (0.26-283.77) | 373 | 18.52  (0.68-274.49) | 57 | 34.46 (0.26-283.77) | 316 | 0.5058 |

** P < 0.05*

**Supplemental table 2. Laboratory characteristics of COVID-19–related hospitalization in cancer patients.**

| **Laboratory Values, Median (Range)** | **Admitted for COVID-19** | **Number Analyzed** | **Not Admitted for COVID-19** | **Number Analyzed** | ***P* Value** |
| --- | --- | --- | --- | --- | --- |
| White blood cell count, K/µL | 5.4 (0.1-737.5) | 237 | 6.0 (0.1-310.8) | 538 | 0.0670 |
| Albumin, g/dL | 3.7 (1.7-4.9) | 237 | 4.2 (1.9-5.1) | 517 | 0.2910 |
| Creatinine, mg/dL | 0.91 (0.31-4.93) | 237 | 0.89 (0.24-12.73) | 539 | 0.0629 |
| Absolute neutrophil count, K/µL | 3.48 (0.08-35.18) | 237 | 3.61 (0.02-59.98) | 535 | 0.0112 |
| Absolute lymphocyte count, K/µL | 0.72 (0.01-685.88) | 237 | 1.35 (0.05-304.58) | 535 | 0.1712 |
| Absolute monocyte count, K/µL | 0.48 (0.01-6.72) | 237 | 0.54 (0.01-6.29) | 535 | 0.0396 |
| Absolute CD4+ cell count, cells/µL | 308 (13-1705) | 38 | 452 (7-3396) | 26 | 0.2911 |
| Absolute CD8+ cell count, cells/µL | 347 (17-5754) | 38 | 378 (6-2313) | 26 | 0.9192 |
| Procalcitonin, ng/dL | 0.14 (0.04-55.31) | 227 | 0.12 (0.03-59.93) | 109 | 0.4127 |
| Fibrinogen, mg/dL | 494 (39-1074) | 223 | 411 (75-988) | 98 | 0.1955 |
| Lactate dehydrogenase, U/L | 246 (92-3870) | 233 | 209 (105-4239) | 375 | 0.1698 |
| Alanine transaminase, U/L | 22 (0-1118) | 237 | 21 (0-584) | 514 | 0.5971 |
| Aspartate transaminase, U/L | 29 (9-2438) | 237 | 22 (9-177) | 514 | 0.9228 |
| Total bilirubin, mg/dL | 0.5 (0.3-7.3) | 190 | 0.4 (0.2-3.0) | 439 | 0.3525 |
| Interleukin 6, pg/mL | 39.15 (3.90-92308.80) | 6 | 7.15 (2.20-28.60) | 6 | 0.6639 |
| C-reactive protein, mg/L | 50.41 (0.27-279.49) | 224 | 10.12 (0.26-283.77) | 150 | 0.5058 |

**Supplemental table 3. Laboratory characteristics of cancer patients with or without severe COVID-9.**

| **Laboratory Values, Median (Range)** | **Severe COVID-19** | **Number Analyzed** | **Non-Severe COVID-19** | **Number Analyzed** | ***P* Value** |
| --- | --- | --- | --- | --- | --- |
| White blood cell count, K/µL | 5 (0.1-737.5) | 131 | 6 (0.1-310.8) | 642 | 0.0050 |
| Albumin, g/dL | 3.5 (1.7 -4.6) | 131 | 4.2 (1.8-5.1) | 621 | <0.0001 |
| Creatinine, mg/dL | 0.92 (0.31-4.93) | 131 | 0.24 (0.24-12.73) | 643 | 0.1323 |
| Absolute neutrophil count, K/µL | 3.4 (0.08-35.18) | 131 | 3.62 (0.02-59.98) | 639 | 0.2583 |
| Absolute lymphocyte count, K/µL | 0.71 (0.01-685.88) | 131 | 1.23 (0.05-304.58) | 639 | <0.0001 |
| Absolute monocyte count, K/µL | 0.45 (0.01-6.72) | 131 | 0.54 (0.01-6.29) | 639 | 0.0024 |
| Absolute CD4+ cell count, Cells/µL | 231 (13-1705) | 20 | 402 (1-3396) | 44 | 0.1924 |
| Absolute CD8+ cell count, Cells/µL | 436 (17-5754) | 20 | 325 (6-2313) | 44 | 0.7119 |
| Procalcitonin, ng/dL | 0.18 (0.04-55.31) | 126 | 0.12 (0.03-59.93) | 208 | 0.0003 |
| Fibrinogen, mg/dL | 527 (39-1074) | 126 | 432.5 (75-994) | 194 | <0.0001 |
| Lactate dehydrogenase, U/L | 321 (92-3870) | 129 | 210 (105-4239) | 477 | <0.0001 |
| Alanine transaminase, U/L | 24 (0-1118) | 131 | 21 (0-584) | 618 | 0.0730 |
| Aspartate transaminase,  U/L | 34 (10-2438) | 129 | 23 (9-318) | 618 | <0.0001 |
| Total bilirubin, mg/dL | 0.5 (0.3-6.7) | 109 | 0.4 (0.2-7.3) | 519 | 0.0076 |
| Interleukin 6, pg/mL | 7.4 (3.9-92308.8) | 9 | 8.5 (2.2-28.6) | 3 | 0.740 |
| C-reactive protein, mg/L | 77.63 (0.68-279.49) | 352 | 15.285 (0.26-283.77) | 21 | <0.0001 |

**Supplement table 4. Characteristics of cancer patients with 30-day COVID-19 attributable mortality.**

| Laboratory values/ median (range) | Died due to COVID-19 | Number analyzed | Survived COVID-19 | Number analyzed | P value |
| --- | --- | --- | --- | --- | --- |
| White blood cell count  K/uL | 4.7 (0.1-200.7) | 25 | 5.9 (0.1-737.5) | 735 | 0.0285 |
| Albumin  g/dL | 3.5 (2.3-4.3) | 25 | 4.1 (1.7-5.1) | 714 | <0.0001 |
| Creatinine  mg/dL | 0.85 (0.51-4.93) | 25 | 0.90 (0.24-12.73) | 736 | 0.3911 |
| Absolute neutrophil count  K/uL | 3.1 (0.08-10.33) | 25 | 3.60 (0.02-59.98) | 732 | 0.1503 |
| Absolute lymphocyte count  K/uL | 0.4 (0.01-10.04) | 25 | 1.18 (0.05-685.88) | 732 | <0.0001 |
| Absolute monocyte count  K/uL | 0.31 (0.01-2.01) | 25 | 0.53 (0.01-6.72) | 732 | 0.0016 |
| Absolute CD4+ cell count  Cells/microL | 179 (25-1705) | 6 | 363 (7-3396) | 58 | 0.2841 |
| Absolute CD8+ cell count  Cells/microL | 391 (17-1337) | 6 | 365 (6-5754) | 58 | 0.8628 |
| Procalcitonin  Ng/dL | 0.17 (0.04-36.39) | 22 | 0.12 (0.03-59.93) | 303 | 0.0685 |
| Fibrinogen  mg/dL | 494 (112-1074) | 21 | 475 (75-1049) | 290 | 0.7563 |
| Lactate dehydrogenase  U/L | 444 (94-3870) | 25 | 217 (92-3083) | 568 | <0.0001 |
| Alanine transaminase  U/L | 21 (0-1118) | 25 | 22 (0-584) | 711 | 0.7404 |
| Aspartate transaminase  U/L | 28 (11-2438) | 25 | 24 (9-473) | 711 | 0.1418 |
| Total bilirubin  mg/dL | 0.5 (0.3-6.7) | 24 | 0.4 (0.2-4.1) | 593 | 0.4877 |
| Interleukin 6 | 10.9 (3.9-92308.8) | 3 | 9.4 (2.2-292.7) | 9 | 0.5175 |
| C reactive protein | 91.48 (8.01-200.38) | 21 | 30.47 (0.26-283.77) | 340 | 0.0005 |
